# CRISPR/Cas9 knockout of human arylamine *N*-acetyltransferase 1 in MDA-MB-231 breast cancer cells suggests a role in cellular metabolism

**DOI:** 10.1038/s41598-020-66863-4

**Published:** 2020-06-17

**Authors:** Samantha M. Carlisle, Patrick J. Trainor, Kyung U. Hong, Mark A. Doll, David W. Hein

**Affiliations:** 1 Department of Pharmacology and Toxicology, University of Louisville School of Medicine, Louisville, KY USA; 2 Division of Cardiovascular Medicine, Department of Medicine, University of Louisville School of Medicine, Louisville, KY USA; 3 Applied Statistics, EASIB Department, New Mexico State University, Las Cruces, NM USA; 4 Present Address: Center for Clinical and Translational Science, University of Alabama at Birmingham, Birmingham, AL USA

**Keywords:** Breast cancer, Cancer metabolism

## Abstract

Human arylamine *N*-acetyltransferase 1 (NAT1), present in all tissues, is classically described as a phase-II xenobiotic metabolizing enzyme but can also catalyze the hydrolysis of acetyl-Coenzyme A (acetyl-CoA) in the absence of an arylamine substrate using folate as a cofactor. NAT1 activity varies inter-individually and has been shown to be overexpressed in estrogen receptor-positive (ER+) breast cancers. NAT1 has also been implicated in breast cancer progression however the exact role of NAT1 remains unknown. The objective of this study was to evaluate the effect of varying levels of NAT1 *N*-acetylation activity in MDA-MB-231 breast cancer cells on global cellular metabolism and to probe for unknown endogenous NAT1 substrates. Global, untargeted metabolomics was conducted *via* ultra performance liquid chromatography-tandem mass spectrometry (UPLC-MS/MS) on MDA-MB-231 breast cancer cell lines constructed with siRNA and CRISPR/Cas9 technologies to vary only in NAT1 *N*-acetylation activity. Many metabolites were differentially abundant in NAT1-modified cell lines compared to the *Scrambled* parental cell line. *N*-acetylasparagine and *N*-acetylputrescine abundances were strongly positively correlated (*r* = 0.986 and *r* = 0.944, respectively) with NAT1 *N*-acetylation activity whereas saccharopine abundance was strongly inversely correlated (*r* = −0.876). Two of the most striking observations were a reduction in *de novo* pyrimidine biosynthesis and defective β-oxidation of fatty acids in the absence of NAT1. We have shown that NAT1 expression differentially affects cellular metabolism dependent on the level of expression. Our results support the hypothesis that NAT1 is not just a xenobiotic metabolizing enzyme and may have a role in endogenous cellular metabolism.

## Introduction

Breast cancer is a heterogeneous disease with many underlying genetic transformations that lead to a diseased state. According to the American Cancer Association, 30% of all new cancer cases in women in 2020 will be breast cancer and 1 in 8 women will develop breast cancer in their lifetime^1^. A better understanding of the role NAT1 has in breast cancer would aide in the development of novel treatment strategies and therapeutics.

Human arylamine *N*-acetyltransferase 1 (NAT1) is a phase-II xenobiotic metabolizing enzyme found in almost all tissues. NAT1 can additionally hydrolyze acetyl-Coenzyme A (acetyl-CoA) in the absence of an arylamine substrate using folate as a co-factor^2,3^. Many additional novel roles for NAT1 have recently been identified using breast cancer cell models including regulation of matrix metalloproteinase 9 (MMP9)^4^, providing protection against reactive oxygen species during glucose starvation^5^, and the loss of NAT1 leading to regulation of mitochondria through inhibition of the pyruvate dehydrogenase complex^6^. NAT1 expression varies inter-individually and has been shown to be elevated in several cancers including estrogen receptor-positive (ER+) breast cancers^7–9^. Additionally, multiple studies have shown that inhibition of NAT1, by both small molecule inhibition and siRNA methods, in breast cancer cells leads to decreased invasive ability and proliferation^10^ and decreased anchorage-independent colony formation^11^.

To date however, the exact mechanism by which NAT1 expression affects cancer risk and progression remains unclear (reviewed in^12^). Differences in endogenous acetyl-CoA have been observed between MDA-MB-231 breast cancer cells expressing parental, increased, and decreased levels of human NAT1^11,13^. A previous study investigating the polar metabolome of the same cells has also revealed differences in a number of metabolites, including amino acids and palmitoleic acid^13^. Given these data, we hypothesize differences in NAT1 activity may be contributing to downstream effects on metabolic pathways that utilize acetyl-CoA such as the TCA cycle and fatty acid synthesis and degradation.

As metabolic reprogramming is one of the hallmarks of cancer proposed by Hanahan and Weinberg^14^ and acetyl-CoA plays a central role in metabolism^15^ we have utilized untargeted metabolomics to investigate how different levels of NAT1 affect cellular metabolism in MDA-MB-231 triple negative breast cancer cells. Metabolomics provides a well-suited method to interrogate global changes in cellular metabolism. Many studies have utilized metabolomics to study cellular metabolism alterations in breast cancer in an effort to increase the understanding of the disease process and to identify potential therapeutic targets or diagnostic biomarkers^16–18^. To build upon our previous metabolomics work^13^, we have expanded the scope of metabolites measured from only the polar metabolome to the global metabolome and also expanded the samples to include complete NAT1 knockout (KO) cell lines as well as a cell line that has only one copy of NAT1 knocked-out (expresses half of parental NAT1 activity).

## Materials and Methods

**Figure 1:**
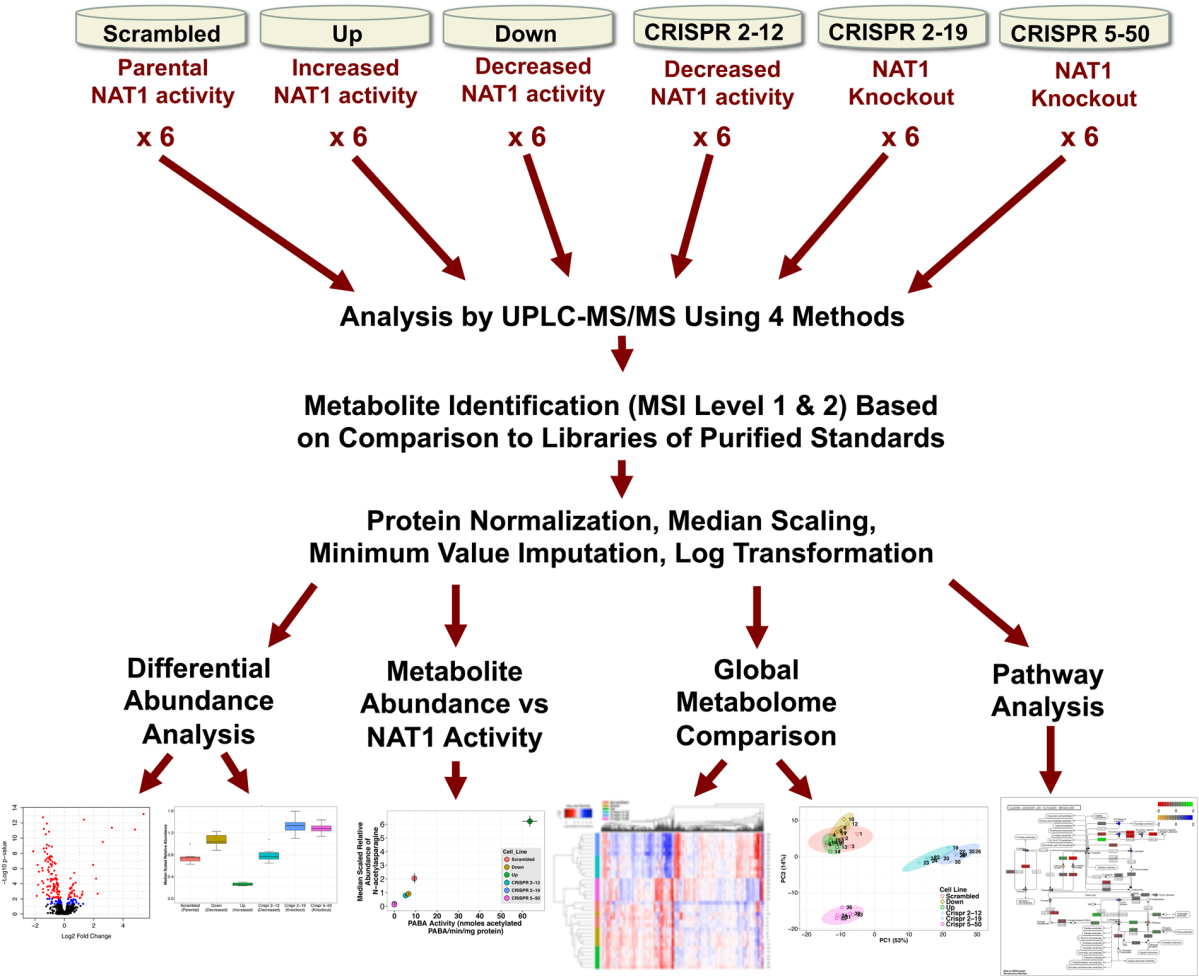
Diagram of Experimental Approach. Six biological replicates from each cell line were collected. Samples were then analyzed by UPLC-MS/MS using 4 methods. Following metabolite identification, abundance data was protein normalized, median scaled, minimum values imputed, and log-transformed. Metabolite abundances were then analyzed for differential abundance, correlation with NAT1 *N*-acetylation activity, unbiased multivariate analysis/clustering, and pathway enrichment.

We conducted a global untargeted metabolomics study on MDA-MB-231 breast cancer cell lines expressing parental (*Scrambled*), increased (*Up*), decreased (*Down, CRISPR 2-12*), or knockout (*CRISPR 2–19, CRISPR 5–50*) human NAT1 *N*-acetylation activity (Fig. 1). The construction and characterization of the *Scrambled*, *Down*, and *Up* cell lines are described in detail elsewhere^11,13^. Briefly, the *Scrambled* cell line was included as a transfection control; the *Up* cell line expresses an approximate 700% increase in NAT1 activity while activity in the *Down* cell line is decreased approximately 40% compared to the *Scrambled* cell line.

Additionally, cell lines constructed using CRISPR/Cas9 technology, expressing approximately 50% of the NAT1 activity of the *Scrambled* cell line (*CRISPR 2–12*) and no detectable (knockout) NAT1 activity (*CRISPR 2–19, CRISPR 5–50*) were included in this study (Fig. 1). The construction and characterization of the *CRISPR 2–19* and *CRISPR 5–50* cell lines has been described elsewhere^19,20^ (*MDA-MB-231 2* = *CRISPR 2–19* & *MDA-MB-231 5* = *CRISPR 5–50* in that manuscript) however the construction of the *CRISPR 2–12* cell line has not been previously described. The *CRISPR 2–12* cell line was constructed using the same methodology and guide RNA as the *CRISPR 2–19* cell line described in Carlisle **et al**.^19^. All cell lines were authenticated by the ATCC Short Tandem Repeat (STR) profiling cell authentication service.

### NAT1 *N*-Acetylation activity assays

*In vitro* NAT1 *N*-acetylation activity was determined in each constructed cell line *via* high performance liquid chromatography (HPLC) using slight modifications of procedures previously described^19,21^. Briefly, cell lysate from each cell line was incubated with 1 mM acetyl-CoA and 300 μM *p*-aminobenzoic acid (PABA) at 37 °C for 10 minutes. Reactions were terminated with the addition of 1/10 reaction volume 1 M acetic acid. Reaction products were collected and analyzed using an Agilent Technologies 1260 Infinity high performance liquid chromatography using a LiChrospher 100 RP-18 (125 × 4 mm; 5 μm) column to determine the amount of acetylated product.

### Collection of Samples

Cells were plated in triplicate per biological replicate at a density of 500,000 cells per 150 × 25 mm cell plate. All cell lines were cultured in high-glucose Dulbecco’s Modified Eagle Medium **(**DMEM), with 10% fetal bovine serum, 1% glutamine, and 1% penicillin/streptomycin added. Cells were allowed to grow for three days in an incubator at 37 °C and 5% CO_2_.

Cells were then harvested on ice by adding 5 mL 0.25% trypsin and scraping the cells from the plate. Three plates were combined to form one sample (biological replicate) to ensure there was enough cell mass for analysis. After harvesting the cells, cells were washed 3 times with ice-cold 1 x phosphate buffered saline (PBS). All supernatant was removed and the cryovials containing cell pellet samples were then flash frozen by placing in a pool of liquid nitrogen for 1 minute followed by immediate storage at −80 °C. Samples were then shipped on dry ice to Metabolon Inc. (Durham, NC) for analysis.

Following receipt by Metabolon, samples were inventoried and immediately stored at −80 °C until processing. Detailed sample preparation, quality assessment/quality control, ultrahigh performance liquid chromatography-tandem mass spectrometry (UPLC-MS/MS), data extraction, compound identification, and metabolite quantification methods are described in detail elsewhere^22^.

### Statistical analyses

Biochemical data was normalized to total protein as determined by Bradford assay to account for differences in metabolite levels due to differences in the amount of material present in each sample. Data for each biochemical was rescaled to set the median equal to 1. Missing values were imputed with the minimum value.

All statistical analyses were performed on median-scaled and log-transformed data using R: A Language and Environment for Statistical Computing version 3.3.1^23^. Data was approximately normally distributed after these transformations. Figure 1 illustrates experimental approach and data analyses methods. One-way ANOVA was performed to test for differences between all groups for each metabolite. *Q*-values were then calculated from un-adjusted *p*-values for preserving the false discovery rate and to account for multiple comparisons^24^. Dunnett’s post-tests were utilized to compare all groups (*Up, Down, CRISPR 2–12, CRISPR 2–19, CRISPR 5–50*) to the *Scrambled* group for those metabolites with one-way ANOVA *q* ≤ 0.05. *Q*-values were also determined and reported for Dunnett’s post-tests.

Fold-change was calculated as previously described^13^. Briefly, the mean abundance for each metabolite was calculated for each group. We then divided the mean of the comparison group (*Up, Down, CRISPR 2-12, CRISPR 2-19, CRISPR 5-50*) by the mean of the *Scrambled* group to give us fold-change relative to the *Scrambled* group. Fold-change and significance of between group differences in global metabolites were visualized using volcano plots. Additionally, abundance data were plotted as box-plots to visualize the distribution of each metabolite by groups. The Pearson correlation coefficient was calculated between NAT1 activity and relative metabolite abundance for all metabolites to generate hypotheses about potential unknown NAT1 substrates or products. Additionally, the correlation coefficient was calculated between carnitine and metabolites whose abundance was concordantly altered in the two NAT1 KO cell lines due to the large proportion of fatty acyl-CoA carnitine conjugates observed. Data was also plotted as a heatmap and hierarchal clustering was conducted using the weighted pair group method with arithmetic mean (WPGMA) method. Principal component analysis was conducted by singular value decomposition of the centered data matrix. The loadings of the first (horizontal-axis) and second (vertical-axis) principal component were plotted. Pathway enrichment analysis was conducted for each group compared to *Scrambled*. The pathway analysis enrichment score was normalized to mean enrichment of random samples of the same size to determine the relative degree of enrichment.

## Results

**Figure 2:**
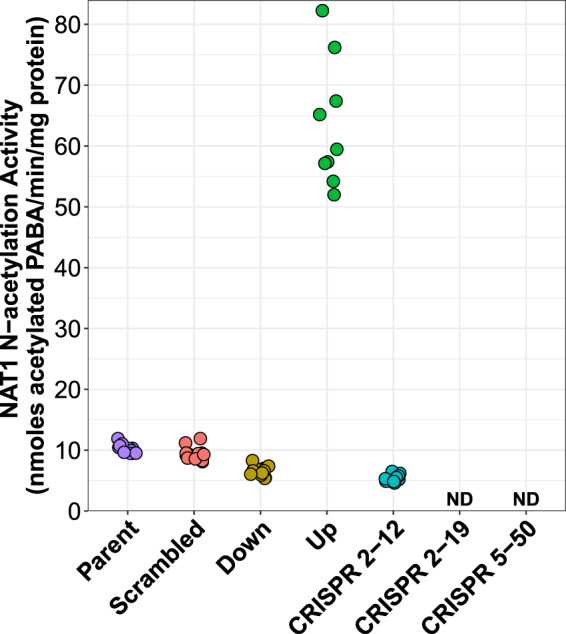
NAT1 *N*-acetylation Activity in the Parent and Constructed MDA-MB-231 Cell Lines. The *Parent* and *Scrambled* cell lines expressed approximately the same level of NAT1 *N*-acetylation activity while NAT1 *N*-acetylation activity in the *Down* and *CRISPR 2-12* cell lines was decreased by approximately 50%. NAT1 *N*-acetylation activity in the *Up* cell line was increased by approximately 700%. The *CRISPR 2-19* and *CRISPR 5-50* had no detectable NAT1 *N*-acetylation activity. Modified from data published previously^19^.

*p*-aminobenzoic acid (PABA) *N*-acetylation activity was measured in each of the six MDA-MB-231 cell lines. The *Scrambled* cell line had approximately the same activity as the *Parent* MDA-MB-231 cell line while the *Up* cell line had an approximate 700% increase in activity. Additionally, the *Down* and *CRISPR 2-12* cell lines had approximately 65% and 50% of the activity of the *Parent* and *Scrambled* cell lines, respectively, while the *CRISPR 2-19* and *CRISPR 5-50* cell lines had no detectable (limit of detection = 0.05 nmoles acetylated PABA/min/mg) activity (Fig. 2).

### Univariate analyses

#### Summary statistics of differentially abundant metabolites

**Figure 3:**
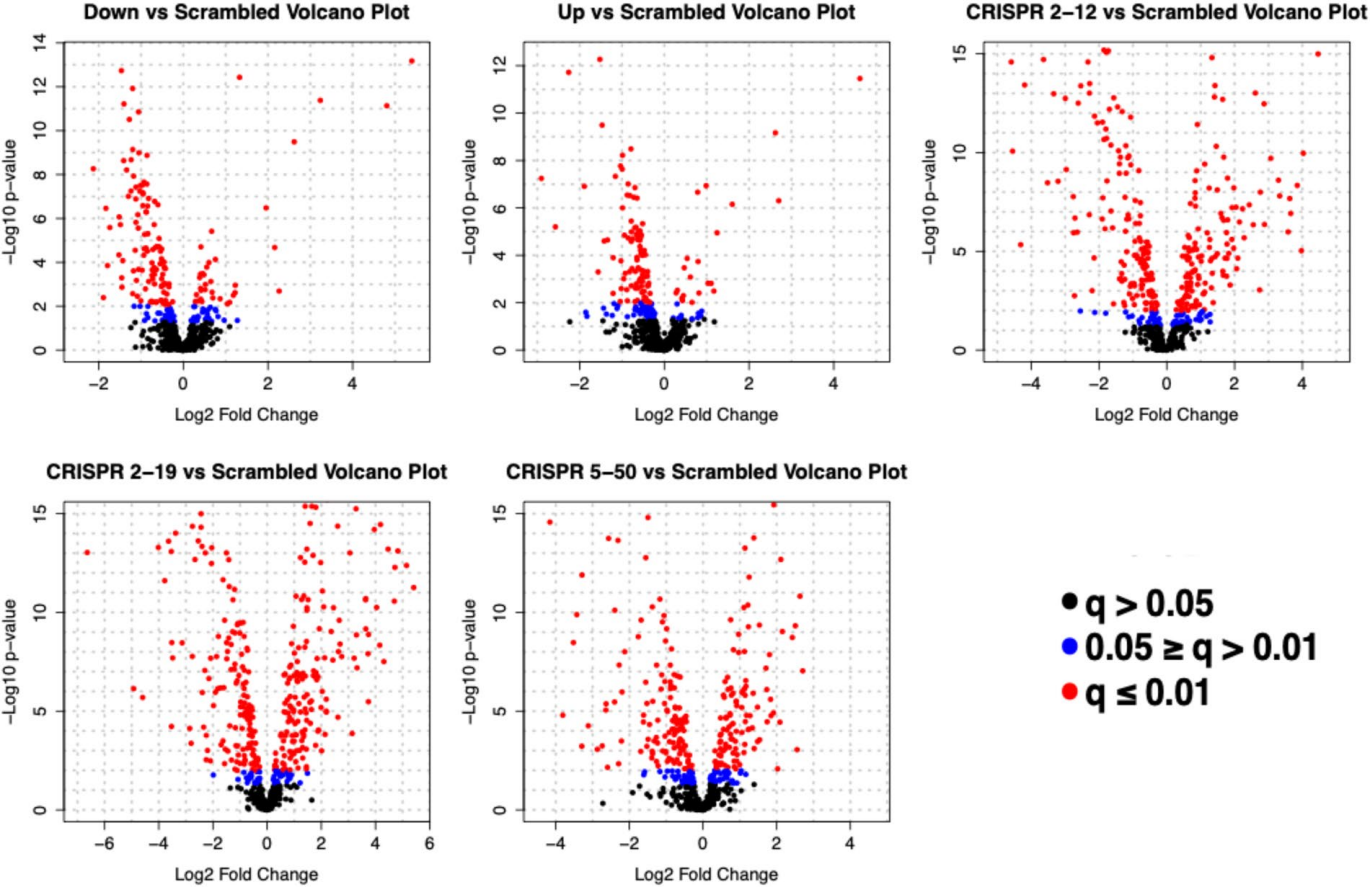
Global Differential Abundance Between All Groups to *Scrambled*. In the volcano plots, each dot represents a single metabolite and is color coded according to q-value. The black dots represent metabolites that had a Dunnett’s post test *q*-value greater than 0.05, blue dots represent metabolites that had a *q*-value less than or equal to 0.05 but greater than 0.01, and red dots represent metabolites that had a *q*-value less than or equal to 0.01. Negative fold changes represent a decrease in that metabolite compared to the *Scrambled* group while positive fold changes represent an increase in that metabolite compared to the *Scrambled* group.

A large proportion (515/567; 90.8%) of the detected metabolites were found to significantly differ (*q* ≤ 0.05) between the six cell lines (Table 1). Following Dunnett’s post-tests it was observed that more metabolites differed in the cell lines constructed *via* CRISPR/Cas9 than the cell lines constructed *via* siRNA when compared to the *Scrambled* cell line. A subset of total detected metabolites (9.5%, 5.6%, 28.4%, 35.8%, and 19.9%) were differentially abundant in the *Down, Up, CRISPR 2-12, CRISPR 2-19*, and *CRISPR 5-50* groups, respectively, with a fold change of 2 or greater (in either direction) compared to the *Scrambled* cell line. Metabolites were further characterized by direction of fold-change compared to *Scrambled* (Table 1; Fig. 3); more metabolites were decreased than increased in all group comparisons to *Scrambled* except the *CRISPR 2-19* cell line. The CRISPR/Cas9 generated cell lines had not only more total metabolites differentially abundant compared to the siRNA generated cell lines, but also more metabolites whose fold-changes were greater than 4.

**Table 1:** Differentially Abundant Metabolites.

One-way ANOVA	Statistical Comparison				
	All Groups				
Total Metabolites p ≤ 0.05	519				
Total Metabolites q ≤ 0.05	515				
Dunnett’s *t*-test Post Test	**Statistical Comparison**				
	***Down*** ***Scrambled***	***Up*** ***Scrambled***	***CRISPR 2-12*** ***Scrambled***	***CRISPR 2-19*** ***Scrambled***	***CRISPR 5-50*** ***Scrambled***
Total Metabolites p ≤ 0.05 FC ≥ 2	56	36	161	206	115
Metabolites (↑↓)	**(15**|**41)**	**(9**|**27)**	**(77**|**84)**	**(121**|**85)**	**(50**|**65)**
Total Metabolites q ≤ 0.05 FC ≥ 2	54	32	161	203	113
Metabolites (↑↓)	**(14**|**40)**	**(8**|**24)**	**(77**|**84)**	**(120**|**83)**	**(50**|**63)**

#### Concordance between differentially abundant metabolites in the two NAT1 Knockout cell lines

**Figure 4:**
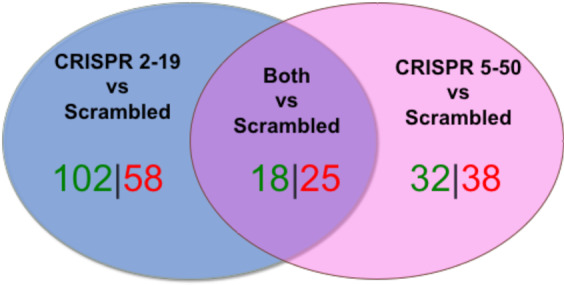
Concordance Between the Two NAT1 Knockout Cell Lines. Significant metabolites with a fold-change greater than or equal to 2 were compared between CRISPR/Cas9 generated NAT1 KO cell lines for concordance. Eighteen metabolites were increased concordantly in the two NAT1 KO cell lines compared to *Scrambled* with 102 and 32 metabolites uniquely increased in the *CRISPR 2-19* and *CRISPR 5-50* cell lines, respectively. Twenty-five metabolites were decreased concordantly in the two NAT1 KO cell lines compared to *Scrambled* with 58 and 38 metabolites uniquely decreased in the *CRISPR 2-19* and *CRISPR 5-50* cell lines, respectively.

**Figure 5:**
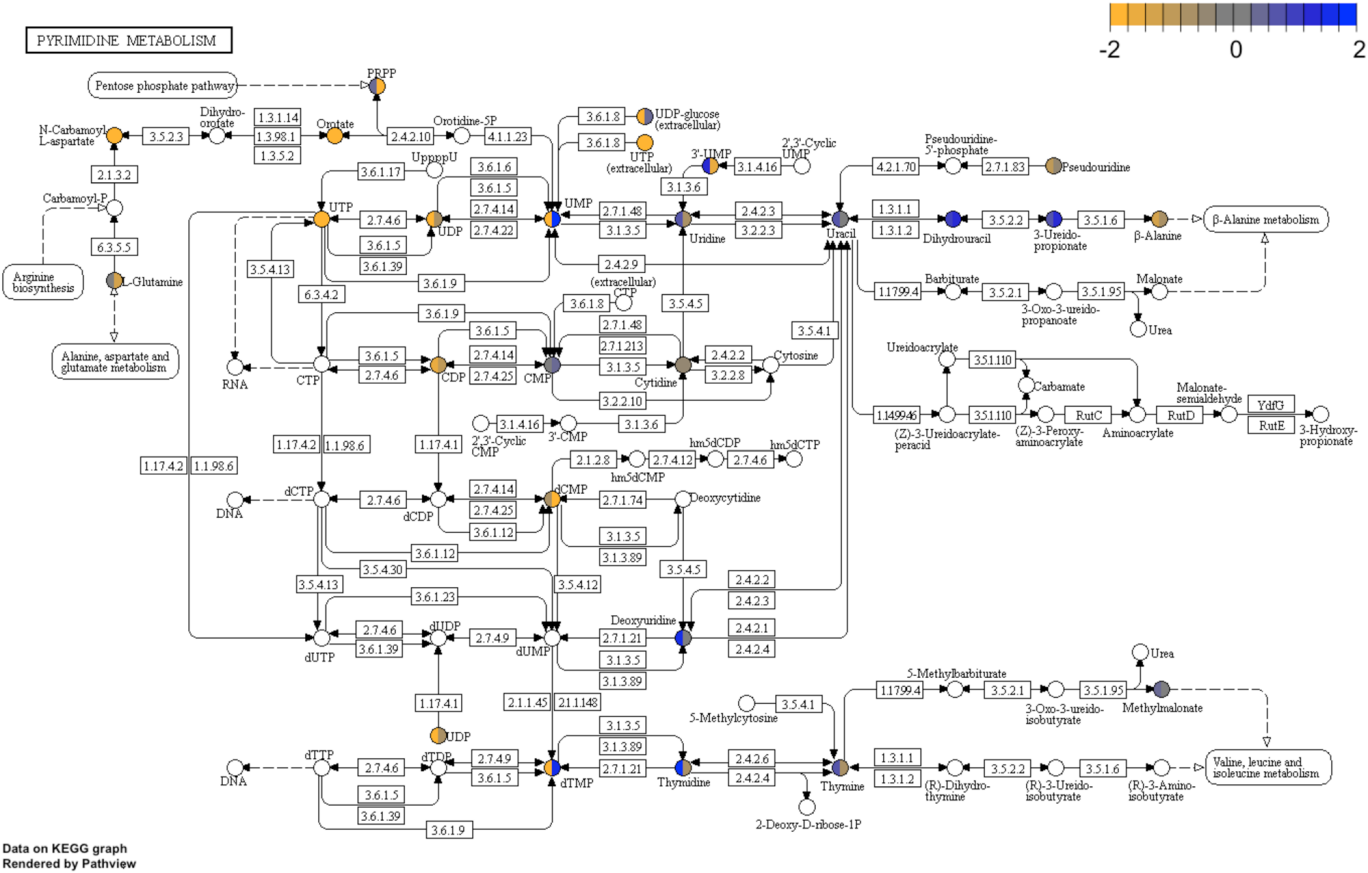
Differential Expression of Metabolites in the Pyrimidine Biosynthesis Pathway in NAT1 Knockout Cell Lines (Compared to *Scrambled*). Metabolite fold-changes in the two NAT1 KO cell lines compared to the *Scrambled* cell line were plotted on the KEGG pyrimidine biosynthesis pathway^25–27^. Each rectangle represents a single gene and each circle represents a single metabolite; the *CRISPR 2-19* cell line fold-change appears on the left half of the circle while the *CRISPR 5-50* cell line fold-change appears on the right half of the circle, Circles not filled represent metabolites for which abundance was not measured.

To ensure we focused on differences related to NAT1 rather than differences related to the specific guide RNA (possible off-target effects) utilized during the knockout of NAT1, the overlap in significant metabolites with a fold-change greater than or equal to 2 was compared between the two NAT1 KO cell lines and the *Scrambled* group (Fig. 4). Eighteen metabolites were increased concordantly in the two NAT1 KO cell lines compared to *Scrambled* with 102 and 32 metabolites uniquely increased in the *CRISPR 2-19* and *CRISPR 5-50* cell lines, respectively. Twenty-five metabolites were decreased concordantly in the two NAT1 KO cell lines compared to *Scrambled* with 58 and 38 metabolites uniquely decreased in the *CRISPR 2-19* and *CRISPR 5-50* cell lines, respectively. Table 2 lists metabolites whose abundances were concordantly changed in the NAT1 KO cell lines. More metabolites had conflicting differential abundance between the two CRISPR NAT1 KO cell lines compared to *Scrambled* that those that agreed. Notably, many of the metabolites decreased concordantly in the CRISPR NAT1 KO cell lines were carnitine conjugates. Assessing correlation between carnitine and the metabolites concordantly changed in the CRISPR NAT1 KO cell lines revealed that the abundance of most of those metabolites were associated with carnitine (Table 2). This suggests differential regulation of carnitine is related to the differential abundances observed in this subset of metabolites. Additionally, many metabolites involved in the pyrimidine biosynthesis pathway were concordantly altered in the CRISPR NAT1 KO cell lines suggesting the knockout of NAT1 affects pyrimidine biosynthesis (Fig. 5).

**Table 2:** Metabolites Concordantly Differentially Abundant in NAT1 Knockout Cell Lines. * indicates compounds that have been tentatively identified.

METABOLITE	BIOCHEMICAL	ANOVA *q*-value	FOLD-CHANGE		Correlation with Carnitine
			*CRISPR 2-19*/ *Scrambled*	*CRISPR 5-50*/ *Scrambled*	
M6	1-(1-enyl-palmitoyl)-2-linoleoyl-GPE (P-16:0/18:2)*	<0.0001	**4.1**	**2.2**	**-0.60**
M26	13-HODE + 9-HODE	0.0010	**2.5**	**2.0**	**−0.68**
M33	1-linoleoyl-GPE (18:2)*	<0.0001	**5.4**	**3.6**	**−0.68**
M116	3-hydroxydecanoate	<0.0001	**2.7**	**3.3**	**−0.91**
M119	3-hydroxylaurate	<0.0001	**2.2**	**2.3**	**−0.77**
M120	3-hydroxyoctanoate	<0.0001	**2.8**	**3.3**	**−0.62**
M233	dihydroxyacetone phosphate (DHAP)	<0.0001	**5.5**	**4.5**	**−0.77**
M339	lactose	<0.0001	**26.4**	**19.1**	**−0.82**
M387	*N*-acetyl-beta-alanine	<0.0001	**13.3**	**4.1**	**−0.72**
M417	nicotinamide ribonucleotide (NMN)	<0.0001	**25.9**	**2.9**	**−0.41**
M437	oleoylcholine	<0.0001	**8.3**	**2.6**	**−0.66**
M445	palmitoleoyl ethanolamide*	<0.0001	**3.0**	**2.3**	**−0.70**
M447	palmitoloelycholine	<0.0001	**8.4**	**2.4**	**−0.57**
M452	palmitoylcholine	<0.0001	**9.7**	**2.1**	**−0.53**
M456	penicillin G	<0.0001	**18.2**	**5.7**	**−0.70**
M481	pyridoxine (Vitamin B6)	<0.0001	**4.5**	**3.8**	**−0.83**
M526	stearoyl ethanolamide	<0.0001	**2.6**	**2.2**	**−0.82**
M555	urate	<0.0001	**2.0**	**3.5**	**−0.87**
M102	2′-O-methylcytidine	0.0019	**0.4**	**0.4**	**0.88**
M110	3-aminoisobutyrate	<0.0001	**0.3**	**0.5**	**0.79**
M132	4-hydroxyglutamate	<0.0001	**0.2**	**0.2**	**0.93**
M161	adrenoylcarnitine (C22:4)*	<0.0001	**0.1**	**0.1**	**0.92**
M176	arachidonoylcarnitine (C20:4)	<0.0001	**0.2**	**0.2**	**0.91**
M190	beta-guanidinopropanoate	<0.0001	**0.3**	**0.4**	**0.84**
M210	cis-4-decenoylcarnitine (C10:1)	<0.0001	**0.2**	**0.4**	**0.69**
M218	cystathionine	<0.0001	**0.2**	**0.1**	**0.92**
M226	cytidine diphosphate	<0.0001	**0.3**	**0.4**	**0.85**
M231	dihomo-linolenoylcarnitine (20:3n3 or 6)*	<0.0001	**0.1**	**0.2**	**0.86**
M232	dihomo-linoleoylcarnitine (C20:2)*	<0.0001	**0.1**	**0.4**	**0.88**
M242	docosatrienoylcarnitine (C22:3)*	<0.0001	**0.1**	**0.2**	**0.88**
M344	laurylcarnitine (C12)	<0.0001	**0.2**	**0.5**	**0.85**
M352	linolenoylcarnitine (C18:3)*	<0.0001	**0.1**	**0.2**	**0.83**
M353	linoleoylcarnitine (C18:2)*	<0.0001	**0.05**	**0.3**	**0.77**
M371	myristoleoylcarnitine (C14:1)*	<0.0001	**0.1**	**0.4**	**0.75**
M376	N2,N2-dimethylguanosine	0.0055	**0.5**	**0.4**	**0.70**
M384	*N*-acetylasparagine	<0.0001	**0.1**	**0.1**	**0.28**
M412	*N*-carbamoylaspartate	<0.0001	**0.1**	**0.1**	**0.89**
M441	orotate	<0.0001	**0.1**	**0.1**	**0.95**
M448	palmitoyl dihydrosphingomyelin (d18:0/16:0)*	<0.0001	**0.4**	**0.4**	**0.95**
M453	pantetheine	<0.0001	**0.5**	**0.3**	**0.78**
M508	sphingomyelin (d18:0/18:0, d19:0/17:0)*	<0.0001	**0.4**	**0.5**	**0.63**
M544	tryptamine	<0.0001	**0.5**	**0.5**	**0.93**
M559	uridine 5′-triphosphate (UTP)	<0.0001	**0.04**	**0.1**	**0.95**

#### Correlation between NAT1 *N*-acetylation activity and metabolite relative abundances

**Figure 6:**
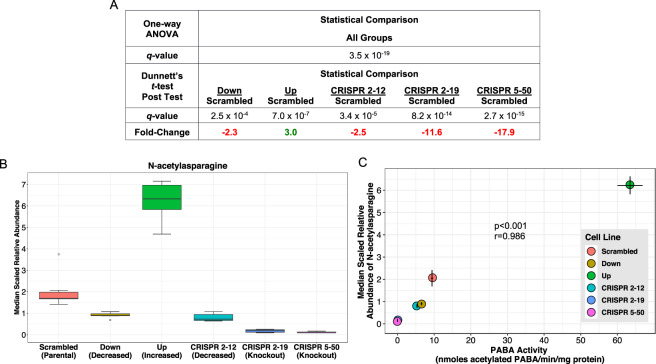
*N*-acetylasparagine Abundance is Strongly Correlated with NAT1 *N*-acetylation Activity. (**A**) One-way ANOVA and dunnett’s post-test *q*-values. (**B**) *N*-acetylasparagine relative abundance by cell line. (**C**) Scatterplot between NAT1 *N*-acetylation activity and *N*-acetylasparagine relative abundance.

**Figure 7:**
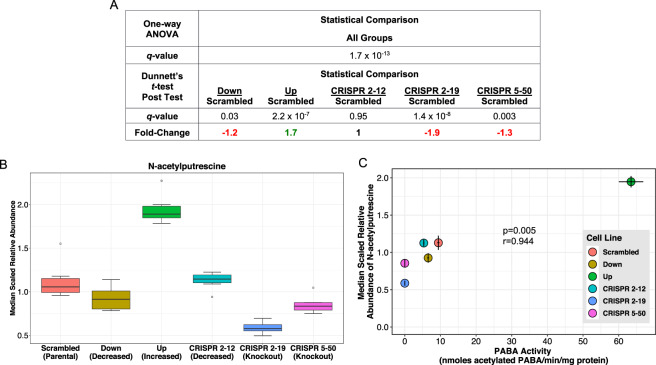
*N*-acetylputrescine Abundance is Strongly Correlated with NAT1 *N*-acetylation Activity. (**A**) One-way ANOVA and dunnett’s post-test *q*-values. (**B**) *N*-acetylputrescine relative abundance by cell line. (**C**) Scatterplot between NAT1 *N*-acetylation activity and *N*-acetylputrescine relative abundance.

**Figure 8:**
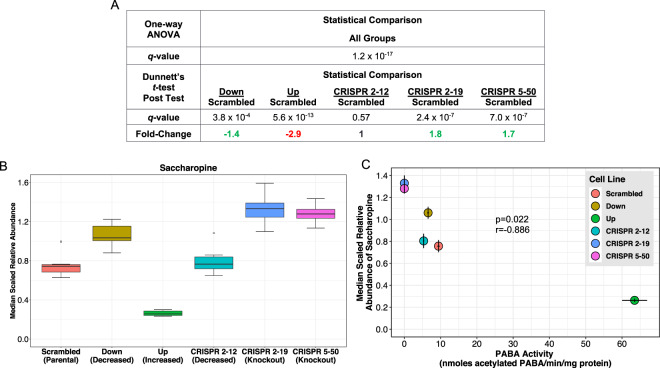
Saccharopine Abundance is Strongly Inversely Correlated with NAT1 *N*-acetylation Activity. (**A**) One-way ANOVA and Dunnett’s post-test *q*-values. (**B**) Saccharopine relative abundance by cell line. (**C**) Scatterplot between NAT1 *N*-acetylation activity and Saccharopine relative abundance.

Eight metabolites, *N*-acetylasparagine, *N*-acetylputrescine, saccharopine, cytidine, 1-palmitoyl-2-alpha linolenoyl-GPC (16:0/18:3n3), isovalerylcarnitine (C5), cysteine sulfinic acid, and serotonin, were significantly associated with NAT1 PABA *N*-acetylation activity (Table 3). The last five of the eight metabolites listed had a high degree of variation in the within-group measurement of metabolite abundance therefore the association is not as well defined as the others. The top two metabolites correlated with PABA *N*-acetylation, *N*-acetylasparagine (Fig. 6) and *N*-acetylputrescine (Fig. 7), had a correlation coefficient (*r*) value greater than 0.90 and are *N*-acetylated compounds, suggesting they may be products of *N*-acetylation by NAT1. Two of the metabolites significantly correlated with PABA *N*-acetylation, saccharopine (Fig. 8) and isovalerylcarnitine (C5), had an inverse relationship suggesting a role in a NAT1 catalyzed reaction as the substrate or possibly down-stream of a NAT1 catalyzed reaction. Additionally, differential acetyl-CoA levels due to NAT1’s ability to hydrolyze acetyl-CoA could be driving these observations.

**Table 3:** Pearson Correlation Between NAT1 *N*-acetylation Activity and Metabolite Relative Abundances. * indicates compounds that have been tentatively identified.

M#	BIOCHEMICAL	CORRELATION COEFFICIENT	*p*-VALUE
M384	*N*-acetylasparagine	0.986	<0.001
M404	*N-*acetylputrescine	0.944	0.005
M491	saccharopine	−0.876	0.022
M224	cytidine	0.856	0.029
M49	1-palmitoyl-2-alpha linolenoyl-GPC (16:0/18:3n3)*	0.825	0.043
M336	isovalerylcarnitine (C5)	−0.820	0.046
M220	cysteine sulfinic acid	0.816	0.047
M498	serotonin	0.811	0.050

### Multivariate/Multivariable analyses

**Figure 9:**
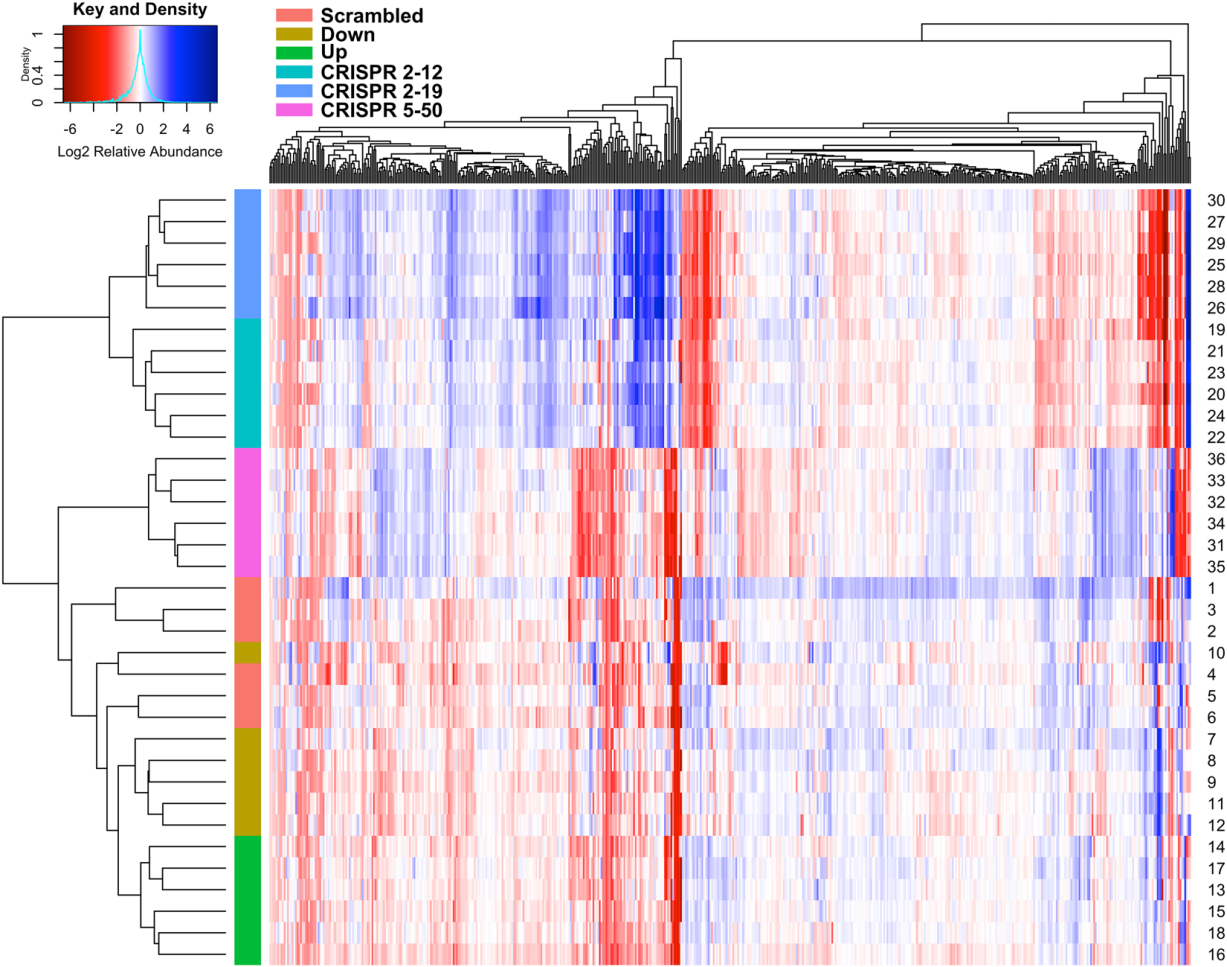
Heatmap with Hierarchical Clustering Indicates Each Cell Line has a Distinct Global Metabolome. Metabolites colored red on the heatmap had a median scaled relative abundance less than 1, metabolites colored white had a median scaled relative abundance of 1, and metabolites colored blue had a median scaled relative abundance greater than 1. Hierarchical clustering reveals the two CRISPR/Cas9 cell lines constructed using guide-RNA 2 are more similar than the two NAT1 complete KO cell lines as would be expected.

Unsupervised hierarchical clustering of each sample revealed a distinct global metabolomic profile of each cell line (Fig. 9). The individual sample replicates clustered accurately by group except for sample 10 of the *Down* group that clustered with the *Scrambled* group. The first split in the dendrogram of the hierarchical clustering is between the two CRISPR/Cas9 cell lines constructed using guide RNA 2 and the other four cell lines; this provides evidence that those two cell lines have global metabolic profiles that are more similar to each other than to the respective cell lines that express the same level of NAT1 *N*-acetylation activity. The heatmap visualization of the data shows distinct clusters of metabolites whose relative abundance is much more similar between the two cell lines constructed using CRISPR/Cas9 guide RNA 2 but have different levels of NAT1 activity than the two CRISPR/Cas9 cell lines that were constructed using two different guide RNAs but had no detectable NAT1 activity.

**Figure 10:**
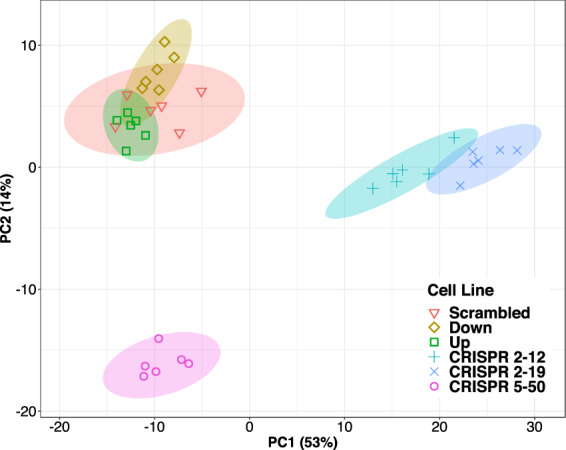
Principal Components Analysis Indicates Each Cell Line has a Distinct Global Metabolome. Each symbol represents an individual metabolomics sample and is color coded by cell line. Principal component one represents 53% of the total variance in our dataset and separates the *CRISPR 2-12* and *CRISPR 2-19* cell lines from all other cell lines. Principal component 2 represents 14% of the total variance in our dataset and separates the *CRISPR 5-50* cell line from all other cell lines.

Similarly, principal component analysis showed that the CRISPR/Cas9 generated cell lines had global metabolomic profiles that were distinct from the siRNA generated cell lines as well as each other. In our dataset, principal component 1 explains 53% of the variance in the data while principal component 2 explains 14% of the variance (Fig. 10). The *CRISPR 2-12* and *CRISPR 2-19* groups are separated from the other four groups by principal component 1. This reveals that these two groups have global metabolomics profiles that are similar to each other but very different from the other four groups given that PC1 represents 53% of the variance in our dataset. The *CRISPR 5-50* group is separated from the other five groups along principal component 2.

**Figure 11:**
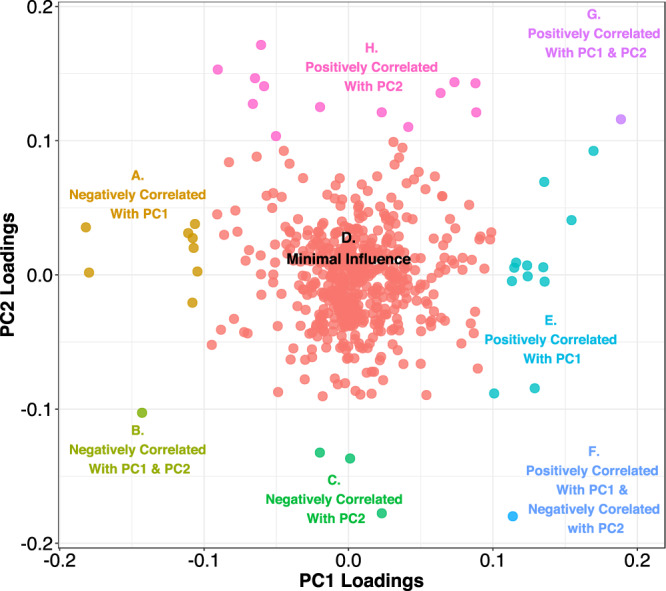
Principal Components Analysis Loadings Plot. Loading from principal component 1 and principal component 2 are plotted showing which metabolites have the greatest contribution to each principal component. Each point represents a single metabolite and is color-coded by contribution to principal component.

From the loadings of each principal component we can infer which metabolites, together, contribute the most to the separation between the groups and thus the variance between the groups (Fig. 11). Most metabolites in the dataset are not contributing to the variance observed. There are groups of approximately 5-15 metabolites that are contributing the greatest to each principle component (in each direction). Adenosine and carnitine related metabolites are negatively correlated with principal component 1 (region A in Fig. 11) while phosphate related metabolites are positively correlated (region E in Fig. 11). Lactate related metabolites are negatively correlated with principal component 2 (region C in Fig. 11) while phosphate and carnitine related metabolites are positively correlated (region H in Fig. 11).

### Pathway analysis

**Figure 12:**
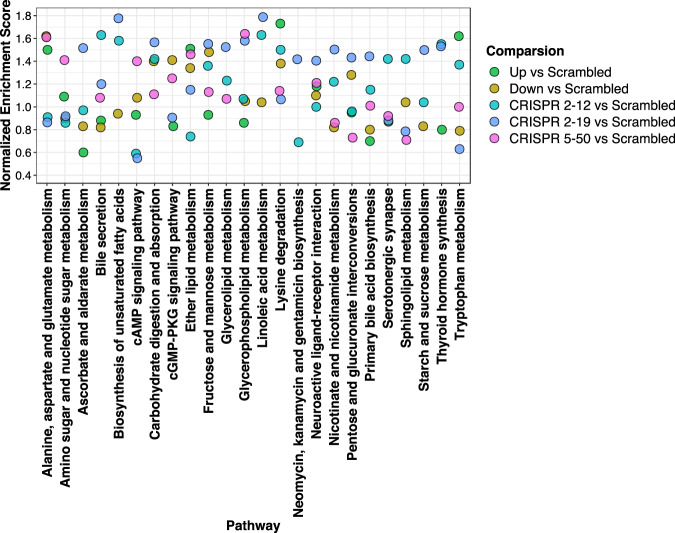
Pathway Analysis Reveals Enrichment of Amino Acid, Lipid, and Fatty Acid Metabolism Pathways. Pathway enrichment analysis was conducted for each group compared to *Scrambled* and is color-coded by comparison. We utilized the normalized enrichment score to determine the relative degree of enrichment.

Pathway enrichment analysis was conducted on each group compared to the *Scrambled* group (Fig. 12). We focused on pathways that had at least one comparison with a normalized enrichment score of >1.20. The Kyoto Encyclopedia of Genes and Genomes (KEGG) pathways were used^25–28^. Disease associated pathways were removed from the analysis results. Amino acid, lipid, and fatty acid metabolism pathways were found to be significantly enriched. Some enriched pathways did not include all group comparisons suggesting differential impacts on metabolism.

## Discussion

Given that we (theoretically) only genetically altered a single gene, NAT1, in each cell line, we expected only a small proportion of metabolites to be significantly different, due to the vast homeostasis mechanisms present^29–31^. However, we observed a very large proportion (~90%) of all metabolites detected to be altered. Additionally, we expected very few, if any, differences in metabolite abundance between the two NAT1 KO cell lines since each cell line should have the exact same genome. Yet one of the most striking observations of this study is the differences in relative metabolite abundance between the two complete NAT1 KO cell lines. The hierarchical clustering, principal components analysis, and pathway enrichment analysis show there were significant differences between the two cell lines constructed using CRISPR/Cas9 guide RNA 2 and the cell line constructed using CRISPR/Cas9 guide RNA 5. Even though, in terms of NAT1 activity the *CRISPR 2-19* and *CRISPR 5-50*, cell lines are highly similar, their metabolic profiles are extremely different. This suggests there may be additional genetic differences between the two cell lines. The observation of a large number of differentially abundant metabolites between each KO cell line compared to the *Scrambled* cell line and compared to each other could be because the cell lines have undergone additional unique mutations between them and/or because each CRISPR guide RNA used caused unique off-target effects in addition to targeting NAT1. To focus on metabolome differences associated with varying levels of NAT1 in breast cancer, we have focused our interpretation of this study to metabolites that agreed between the two CRISPR NAT1 KO cell lines (Table 2).

A striking finding from the present study is that NAT1 KO cells seem to have defective β-oxidation of fatty acids. A vast majority of the metabolites that are found to be reduced in both NAT1 KO cell lines belong to acylcarnitine species, including laurylcarnitine (C12), linolenoylcarnitine (C18:3), linoleoylcarnitine (C18:2), and myristoleoylcarnitine (C14:1) (Table 2). Formation of acylcarnitines which is performed by carnitine palmitoyltransferase 1 (CPT1) is the first and crucial step in transport of fatty acids into mitochondria. Interestingly, malonyl-CoA, which is a known regulator of fatty acid synthesis and oxidation^32^, is formed by carboxylating acetyl-CoA and is known to inhibit CPT1. Decreases in the level of a number of acylcarnitine species clearly indicate that the CPT1 step and subsequent mitochondrial metabolism of fatty acids are disturbed in NAT1 KO cells. We hypothesize the increase in acetyl-CoA observed when NAT1 is knocked out may lead to increased malonyl-CoA which inhibits CPT1 resulting in less acylcarnitines. In addition, 3 out of 18 metabolites that are concordantly increased in both KO cell lines belong to 3-hydroxy-fatty acids (e.g., 3-hydroxydecanoate, 3-hydroxylaurate, and 3-hydroxyoctanoate) (Table 2) which are intermediates in β-oxidation of fatty acids. The reduced levels of acylcarnitine species and accumulation of 3-hydroxy-fatty acids, together, strongly suggests that both transport of fatty acids into mitochondria as well as metabolism of fatty acids are compromised in NAT1 KO cells. This suggests the involvement of NAT1 in fatty acid metabolism. Another possible explanation is that the defects in fatty acid metabolism may reflect mitochondria dysfunction.

To further clarify whether this observation was due to dysregulation of fatty acyl-CoA species or carnitine in NAT1 KO cell lines, we accessed association between carnitine and metabolites decreased and increased concordantly in both NAT1 KO cell lines. Nearly every metabolite concordantly differentially abundant showed strong correlation with carnitine suggesting the observation may follow the differential regulation of carnitine. Carnitine is biosynthesized from lysine and methionine^33^ with important biological roles in the transport of activated long-chain fatty acids from the cytosol to the mitochondrial matrix for β-oxidation^34,35^, modulation of the acyl-CoA/CoA ratio^35,36^, and storage of energy as acetylcarnitine^36,37^. Further studies are required to discern whether these observations are due to NAT1 directly or indirectly due to NAT1’s effect on acetyl-CoA levels or other possible indirect effects.

One of the most noticeable findings that emerged from the current metabolomics data was a reduction in *de novo* pyrimidine biosynthesis in the absence of NAT1. This was indicated by significant and concomitant decreases in the level of different intermediates that are involved in the pyrimidine biosynthetic pathway, including N-carbamoylaspartate, orotate, UTP, and CDP, in both NAT1 KO cell lines (Table 2). Moreover, cytidine (a ribosyl pyrimidine) was among the few metabolites whose levels were positively correlated with NAT1 activity (Table 3). Taken together, these indicate that the cellular level of pyrimidine biosynthetic intermediates as well as cytidine may be either directly or indirectly regulated by NAT1 activity. Pyrimidine nucleotides play a critical role in cellular metabolism^38,39^. They serve as activated precursors of RNA and DNA. In addition, CDP-diacylglycerol is a key intermediate in the synthesis of phospholipids, and UDP-sugars serve as sugar donors in protein glycosylation. As such, *de novo* pyrimidine biosynthesis is indispensable during cell growth and proliferation to meet the demand for nucleic acid precursors and other cellular components. Accordingly, the *de novo* pyrimidine biosynthetic pathway is invariably upregulated in rapidly growing neoplastic cells^40^. We and others have previously reported that depletion of NAT1 in cancer cell lines often leads to a reduction in growth potential under specific conditions^10,11,20,41,42^. Although speculative, it is plausible that the deficiency in cellular pyrimidine pool may contribute to the growth delay observed in NAT1 KO cells. Although the mechanism by which NAT1 depletion leads to an alteration of the pyrimidine biosynthetic pathway is currently unclear, the present observation has an implication in breast cancer therapy. Recently, Brown and colleagues have reported that resistance to chemotherapy in triple-negative breast cancer cells can be circumvented by inhibiting the *de novo* pyrimidine synthesis pathway^43^. In addition, targeting *de novo* pyrimidine synthetic pathway has been shown to be a successful strategy in treating different types of tumors^44–47^. Based on this finding, targeting and inhibiting NAT1 expression or activity may increase the sensitivity of breast cancer to chemotherapeutic agents.

The strong positive association between the abundance of *N*-acetylasparagine and NAT1 activity in the 6 MDA-MB-231 cell lines is consistent with *N*-acetylasparagine being a product of NAT1 *N*-acetylation. There is currently no known mechanism or enzyme responsible for the acetylation of free amino acids. It is hypothesized that acetylated free amino acids originate from the breakdown of *N*-terminal acetylated proteins however L-asparagine residues in proteins are not known to be acetylated by NAT1^48^. Additionally, the biosynthesis of *N*-acetylasparagine is not defined in the literature. Recombinant murine and human aminoacylase 2 (ASPA) have been reported to catabolize *N*-acetylasparagine, albeit at a much lower level than the prototypic ASPA substrate *N*-acetylaspartate^49^. Additionally, aminoacylase 1 deficiency (ACY1), a rare inborn error of metabolism disease, is diagnosed by increased *N*-acetylated amino acids in the urine, including *N*-acetylasparagine^50^ suggesting ACY1 can also metabolize *N*-acetylasparagine. Given the abundance distribution of *N*-acetylasparagine in our constructed cell lines, it is possible that L-asparagine is a substrate *N*-acetylated by NAT1 to form *N*-acetylasparagine but confirmation is needed.

Similarly, the strong positive association between the abundance of *N*-acetylputrescine and NAT1 *N*-acetylation activity in the 6 MDA-MB-231 cell lines is consistent with *N*-acetylputrescine as a product of NAT1 *N*-acetylation. Putrescine is known to be *N*-acetylated by spermidine/spermine N1-acetyltransferase 1 (SAT1) and spermidine/spermine N1-acetyltransferase 2 (SAT2) but with much lower affinity than for other SAT substrates such as spermidine^51^. Redundancy in non-homologous metabolic enzymes has been shown to occur^52,53^ and it is possible that NAT1 and the SATs may both *N*-acetylate putrescine.

Both L-asparagine and putrescine have been implicated in the promotion of cell growth^54–59^. Our data indicates cell lines with higher levels of NAT1 activity have increased amounts of *N*-acetylated asparagine and putrescine while cell lines with decreased and knocked-out NAT1 activity have decreased amounts of *N*-acetylated asparagine and putrescine. We hypothesize this could lead to decreased asparagine and putrescine in cell lines with high NAT1 and increased asparagine and putrescine in cell lines with decreased and knocked-out levels of NAT1 relative to cell lines with basal NAT1 activity. However, we did not observe this differential distribution in our dataset. It has been shown that intracellular asparagine exchanges with extracellular amino acids to promote mTORC1 activation, protein and nucleotide synthesis and cell proliferation under normal growth (non-starvation) conditions^55^. Additionally, it has been reported that the invasiveness of a mouse breast cancer model could be modulated either by altering asparagine biosynthetic capacity or by modifying extracellular asparagine pools with decreases in asparagine leading to decreased metastatic burden^54^. Polyamines, such as putrescine, are known to facilitate the interactions of transcription factors, such as estrogen receptors with their specific response element and are involved in the proliferation of ER negative breast cancer tumor cells (reviewed in^60^). Additionally, an increase in intracellular polyamine concentrations has been associated with increased cell proliferation and has been linked to tumorigenesis^61–65^. Our data, interpreted in light of the results of previous studies, suggests that knockout NAT1 may increase cell growth capabilities due to higher levels of asparagine and putrescine however studies from our lab do not support this^20^. This may be because there are additional mechanisms in place regulating the amount of free asparagine and putrescine or other factors regulating growth that override an abundance of these metabolites.

The strong negative association between the abundance of saccharopine and NAT1 activity in the 6 MDA-MB-231 cell lines suggests saccharopine is a NAT1 substrate or located upstream of a NAT1 catalyzed reaction. Saccharopine is an intermediate in the main pathway responsible for the catabolism of lysine^66,67^. This observation may be connected to lysine’s role in carnitine biosynthesis^33^. Pathway analysis indicated pathways that directly involve acetyl-CoA such as the lysine degradation and tryptophan metabolism pathways were enriched for differences (Fig. 12). Additionally, pathways that feed into the TCA cycle were also significantly enriched suggesting NAT1 has an impact on energy metabolism.

## Conclusions

Our results support the hypothesis that NAT1 is not just a xenobiotic metabolizing enzyme and may have a role in endogenous cellular metabolism. Whether this is direct or through NAT1’s effect on acetyl-CoA remains unknown. We have shown that NAT1 expression differentially affects cellular metabolism dependent on the level of expression. Additionally, we have identified potential novel substrates and products of *N*-acetylation by NAT1 but further validation is required. These metabolites have recently been implicated in enhanced cell growth and metastatic potential in breast cancer models suggesting they may be the key to understanding how varying levels of NAT1 affect breast cancer progression. Furthermore, metabolites involved in fatty acid metabolism and pyrimidine biosynthesis showed strong concordant changes in both NAT1 KO cell lines suggesting NAT1 may have an essential role in these processes. Moreover, many of the pathways significantly enriched in pathway analysis are pathways where acetyl-CoA plays a role, adding further evidence for the connection between NAT1 and acetyl-CoA in metabolism.

## Supplementary information

Supplementary information.

Supplementary information2.

## Data Availability

Processed metabolite relative abundance data is available as a Supplementary File. Reasonable requests for the transformed MDA-MB-231 cell lines utilized in this study will be considered.
