# Supplementary material for: CRISPR/Cas9 knockout of human arylamine N-acetyltransferase 1 in MDA-MB-231 breast cancer cells suggests a role in cellular metabolism
Source: Sci Rep. 2020 Jun 17;10:9804. doi: 10.1038/s41598-020-66863-4 (PMC7299936; doi:10.1038/s41598-020-66863-4)
Supplement: Supplementary file 2 — Supplementary information2. [file 41598_2020_66863_MOESM2_ESM.docx]

CRISPR/Cas9 knockout of human arylamine *N*-acetyltransferase 1 in MDA-MB-231 breast cancer cells suggests a role in cellular metabolism

*Samantha M Carlisle*^1^*, Patrick J Trainor*^2,3^*, Kyung U Hong*^1^*, Mark A Doll*^1^*, David W Hein*^1*^

^1^Department of Pharmacology and Toxicology, University of Louisville School of Medicine, Louisville, KY
^2^Division of Cardiovascular Medicine, Department of Medicine, University of Louisville School of Medicine, Louisville, KY
^3^Applied Statistics, EASIB Department, New Mexico State University, Las Cruces, NM

KEYWORDS: Human Arylamine *N*-Acetyltransferase 1, NAT1, Breast Cancer, Metabolomics, MDA-MB-231

^*^Corresponding Author
